# Some Suggestions in Using Bromides in Epilepsy1Read at the thirty-third annual meeting of the Medical Association of Central New York, held at Rochester, N. Y., October 16, 1900.

**Published:** 1901-02

**Authors:** L. Pierce Clark

**Affiliations:** First Assistant Physician, Craig Colony for Epileptics, Sonyea, N. Y.


### BUFFALO MEDICAL JOURNAL.

VOL. XL.—LVI. FEBRUARY, 1901. No. 7.

### Original Communications.

### SOME SUGGESTIONS IN USING BROMIDES IN EPILEPSY.[1]

BY L. PIERCE CLARK, M. D.,

First Assistant Physician, Craig Colony for Epileptics, Sonyea, N. Y.

SINCE the introduction of the bromide salts in 1851, by Locock and McDonnell, their use in the treatment of epilepsy has had a varying fortune. Some maintain that they should be used only after all other remedies have been tried, and others still claim that they are really the only remedies we have for the disease. Between these two extremes of opinion we have varying views. Undoubtedly the perfunctory administration of bromides has been productive of great harm. The train of their evil effects is too well known for me to detail them; instead, I propose to give some experience of my own with the bromide salts.

I hope it will be understood at the outset that I regard the bromides as but one of the many drugs to be used in recovering epileptics from their disease. The modern conception of the disease demands that much be done unremittingly for the possible amelioration of the predisposition which really underlies all the epilepsies. All agencies for the physical and mental regeneration of the epileptic must be evoked in every case. I regard bromides as one of our great drug adjuvants for the curative treatment of the disease. Until the seizures are stopped we cannot hope to successfully invoke other far-reaching and more telling methods. Above all other considerations, the seizures must be stopped; bromide is the drug par excellence for this phase of the disease. The cortical stability of the epileptic once

1. Read at the thirty-third annual meeting of the Medical Association of Central New York, held at Rochester, N. Y., October 16, 1900.

assured, many things are then possible. With this prefatory remark on my position, I will take up the title of the paper.

Epileptics and their relatives expect physicians to give medicines at once which will lessen the frequency and severity of the spasms; therefore, too little attention is generally given in the beginning to a careful case study of the digestive tract and its tolerance of the bromides. If the patients are anemic and have the epileptic physique or dyscrasia, we must try to build up the organism while administering bromides. I almost always give iron tonics and cod liver oil to epilpetics, either to correct existing mal-nutrition or forestall the effects of the bromides. I consider this adjuvant treatment as important to the bromides as the diet and the general living regimen. The bromides may be given at once. I use the following well-known formula, which I am persuaded is the best:

ELIXIR OF THE TRIPLE BROMIDES.

℞ Potass. bromid.
Ammon. bromid.
Sodii bromid. . . . . . . . . . . . . . . . . . . . . . . āā gr. v.
Elixir simplex, q. s. ad. . . . . . . . . . . . . . . . . ℨi.
M.

This preparation is given because it is palatable and contains the dynamical effect of 15 grains of bromide to the drachm, an easy and convenient unit to work upon in further bromide treatment. The patient is begun on one drachm a day at the outset, given night or morning, according to whichever end of the day the seizures occur most frequently. This is kept up for a week or so, until the sedative power of the single dose can be determined, and then successive drachms are added as thought necessary, until the seizures cease or 480 grains or one ounce of the salt is taken. The giving of bromides of late has been in too small doses to gain antispasmodic effect. The amount should be sufficient to stop the seizures. The same care and attention should be exercised in giving bromides in the different cases as though we were handling a new drug each time.

Every possible precaution in the prevention of bromide intoxication must be taken. The epileptic organism must be kept in the highest possible functionating state. Hot and cold baths, massage and electricity should be given. If the patient has a good physique, I order a prolonged hot bath twice each week on retiring. I order a cold shower bath and a friction rub every morning on rising. If the patients cannot stand the shock, a cup of coffee or hot milk is given them in bed before the bath, and if the shock is still too severe they should lie down until the reaction takes place; a hot drink after a cold bath materially aids the rapid reaction. No alcoholic stimulants, as a rule, should be allowed in connection with these cold shocks. Put just as little dependence on hot drinks as possible, and insist upon neurotic patients themselves making efforts to favor the reaction. I regard these cold baths in the morning as vital to maintenance of a good peripheral circulation, to physiological awakening of the organic tissues.

The bowels must be watched, the diet must be prescribed; high rectal saline douches should be given once or twice each week. The mouth must be kept scrupulously clean. Internal antiseptics should be given at stated intervals, and if the face shows the effects of the bromide intoxication, cloths wrung out of hot water may be applied to the face to relax the tissues and aid the circulation in taking up the bromide deposits. This skin treatment should be followed nightly for fifteen to twenty minutes; the last moist heat application should be a hot saturated solution of boracic acid, and a final application of zinc ointment should end each night's treatment to the face. I have never seen a case of acute or chronic bromism of the skin which would not to a great extent yield to this treatment, when the other factors in bodily hygiene were faithfully looked after. It is absurd to look to Fowler's solution of arsenic to remove bromide intoxication when the care of administering bromide is neglected.

Having stopped the seizures by large doses of bromide, the work of treating the epilepsy has only begun. The dosage must be maintained for as long a time as possible without intoxication. Only so much bromide is effective as the tissues will absorb and hold; the longer time the greater amount they can be made to maintain the better. Not infrequently the patient is left to himself at this stage of bromide treatment, and he soon disregards the laws of hygiene, and bromide intoxication follows swiftly. The seizures occur more frequently than before and the patient's life is saved by some physician withdrawing the bromides. Cases in which the care of administering bromides is neglected, form no small part of those said to stand the bromides poorly. Epileptics taking bromides in large doses should be under the careful supervision of some competent person, and be seen by a physician at frequent intervals. While establishing the dosage for any particular case, the physician should see the patient two or three times each week; every day is better. After the individual regimen is established thoroughly, the patient may go two or three months without a consultation. Not infrequently after the bromide dosage has been adjusted to the seizure level, epileptics will suddenly surprise the physician by having two or three seizures just at the active or second phase of treatment is begun. The explanation is simply this: the patient, from being a passive agent, from living a sedentary life, is started on an active out-of-door life for the establishment of a real and permanent cure if possible. The patient's bodily activity increases and hastens the elimination of the bromide salts, and the seizures recur. Add 15 to 30 grains to the dose in changing the patient from a sedentary to an active out-of-door life.

Of late when cases will not stand the bromide salts in high dosage, I give bromide in the following simple formula:

TEN PER CENT. BROMINE.

| | | |
|---|---|---|
| ℞ | Ol. sesamum . . . . . . . . . . . . . . . . . . . . . . . | ℥ix. |
| | Bromine, pure . . . . . . . . . . . . . . . . . . . . . . | ℥i. |

M. S.—℥ss. night and morning; increase as directed.

Or, in this emulsion, which I have found both more highly nutritive and sedative:

EMULSION OF BROMINE.

| | | |
|---|---|---|
| ℞ | Ol. sesamum . . . . . . . . . . . . . . . . . . . . . . . | ℥viii. |
| | Gum. acacia gran. . . . . . . . . . . . . . . . . . . . . | ℥ii. |
| | Syr. simplex . . . . . . . . . . . . . . . . . . . . . . | ℥ii. |
| | Ol.Gaultheria . . . . . . . . . . . . . . . . . . . . . . | mlx. |
| | Aqua . . . . . . . . . . . . . . . . . . . . . . . . . | ℥vi. |

M. Ft.—Emulsio. Add bromine, pure, gr. dcccclx. S.—℥ss. night and morning, increase as directed.

(Sample of emulsion submitted.)

Or it may be combined in this emulsion with potassium bromide, in order that the pure bromine will not be lost by fuming. The vegetable oil may be changed for pure Norwegian cod liver oil:

EMULSION OF BROMINE, MODIFIED.

| | | |
|---|---|---|
| ℞ | Gum. acacia gran. . . . . . . . . . . . . . . . . . . . . | ℥ii. |
| | Ol. sesamum or ol. morrhuæ . . . . . . . . . . . . . . | ℥viii. |
| | Aqua . . . . . . . . . . . . . . . . . . . . . . . . . | ℥vi. |
| | Ol. Gaultheria . . . . . . . . . . . . . . . . . . . . . | mlx. |

M. Ft.—Emulsio. Add bromine, pure, grs. dcccclx. Potass. bromide, grs. cccclxxx. ℥ss. bromine, grs. xxx. Potass. bromide, grs. xv. S.—℥ss. night and morning, and increase as directed.

(Sample of emulsion submitted.)

Bromine given in this manner is less irritating to the intestinal tract, is not constipating, is markedly less toxic, and seems to be much more lasting in its effects. It should be given a more extensive trial, especially where the single or combined bromide salts fail. In making the bromine mixture the druggist needs to be quick in adding the bromine to prevent loss from fuming. In giving bromine, please consider 1 gr. by weight of bromine as equal to 2 grs. of bromide salts from a therapeutic standpoint.

I have reserved to the last to speak of what I regard as without doubt the greatest adjuvant to the bromides discovered in recent years. It is a method which I am now using successfully on several epileptic patients, and which is known technically as hypochlorisation or salt starvation. This method was brought forward by Toulouse last year, who reported a series of very successful trials and more recently several continental physicians have reported favorably. Its rationale is simply that bromine can substitute chlorine in the tissue. Experiments upon dogs and cats fed for ten days upon bromine, showed that more bromine could be procured from their remains than chlorine. In the kidneys and bone marrow the proportion was two to one. Bromine is not a foreign substance, but a true mineral constituent of the body; when discontinued it is replaced by alimentary chlorine and requires about four months for its complete elimination. It is therefore evident that bromine may partly replace chlorine chemically and physiologically as well. Linossier, a French neurologist, in investigating the subject upon animals found that in the gastric juice, the chlorhydric acid could be replaced by bromhydric acid. Thus, it is seen that we have a good basis for the clinical fact that when an epileptic is under salt starvation, the bromides are more greedily absorbed in the tissues. It is always the amount of bromides which the tissues will hold that determines the efficacy of the bromide medication; therefore we can at once see how very important this method is in the treatment of epilepsy. Nearly all epileptics are very fond of salt, and eat large quantities of it, and in consequence it is difficult to get many of them to undergo voluntary deprivation of salt. So a semi-starvation of salt is about all that can be expected from them. One of the best and easiest ways of carrying out this adjuvant of the bromides, is the establishment of a milk diet with occasional removals to a mild salt diet. For a long time it has been known that a milk diet assists much in lessening the formation of toxic substances in the alimentary tract. To aid the carrying out of the salt starvation treatment, in which a relapsing diet is necessary, or an accompanying semi-starvation diet is desired, I have modified Toulouse's daily dietary so as to give the following:

BREAKFAST.—Milk, 1 pint.
LUNCH.—Two cakes made of eggs, farina, milk and sugar; coffee.
DINNER.—Bouillon, unsalted; boiled beef, unsalted; potatoes.
SUPPER—Porridge made with farina, sugar, boiling milk, etc.

By this method we can reduce the usual amount of bromides one-half and still get the same results while lessening the chance of bromide poisoning one-half. The economy of this method is obvious both in epilepsy and other diseases where sedation is needed over a long period of time. Many practical points still remain unsolved in hypochlorisation, but sufficient has been proven to warrant us in giving the method extended trial in connection with the bromide treatment in epilepsy.

These large doses of bromides should be continued for several weeks after the seizures stop; then week by week, reduce the bromides 15 grs. per week, until you get to the seizure level again and let that dosage remain to suppress the convulsions. Then, and not until then, can we consider our patient free fiom the first phase of hospital treatment. Arrangement should now be made for daily exercise, school, manual training, and other occupations of life, preferably in the country and at agricultural pursuits; physical activity should be maintained for several hours daily.

Some illustrative cases under high dosage of bromides may be cited here. The cases were either those that had not been helped by bromides or other medication, or, those in whom bromides were not given to the point of desired therapeutic effect, on account of a rapid and dangerous toxicity developing. Precautions enumerated in this paper were not undertaken heretofore.

Case I.—Girl, 17 years old; idiopathic epileptic; ordinary medication did not influence seizures. Had on an average 250 grand mal and petit mal attacks per month. Bromides were given according to the methods stated in this paper. The seizures were not modified until 200 grs. daily were taken; they then occurred 40 times per month; the next month on 300 grs. they dropped to 4 attacks, and under 480 grs. daily the attacks ceased altogether. Patient has had only a half dozen slight losses of consciousness for the past 18 months. She is now taking 240 grs. daily, and is in excellent bodily and mental health, has grown 5 inches taller, and gained 12 pounds in weight during the past year. Although bromides are counted unhygienic, the seizures in this case must have been very detrimental to growth and development.

Case II.—Traumatic epileptic; trephined. Epilepsy continued and all drugs, including ordinary doses of bromides, failed. Patient had 12 to 14 grand mal seizures per month. Bromides were gradually given until 280 grs. per day were taken. The seizures ceased entirely and have not showed themselves for six months. Patient is improving mentally and physically—is practically a new man. He takes 180 grains daily and shows no bromide intoxication, not even a facial acne.

Case III.—Man, 26 years old, an idiopathic epileptic with psychical seizures consisting of but momentary losses of consciousness; never had convulsions. Epileptic for 20 years; had never found a remedy that could affect the regular periodicity of the psychical series until placed on bromides. He is now on 160 grains daily and has had no attacks whatever for over four months. There has been a very remarkble improvement physically and mentally. Slight facial acne and general bromic toxicity occurred two or three times, but were easily corrected. No one would now know that this patient ever had taken or was now taking bromides by his facial or general appearance.

In conclusion I would summarise as follows:

1. Bromides still hold a very important place in epileptic treatment.

2. Tonics must be given constantly while administering bromides.

3. Bromide salts should be given gradually to find the epileptic's sedative level.

4. Baths, high enemas, alimentary antiseptics, massage and electricity are absolutely essential to successful bromide medication.

5. Bromine is a worthy successor to the bromides in many cases.

6. Salt starvation or semi-salt starvation is a great adjuvant to the bromide treatment.

### DISCUSSION.

Dr. Lester, Seneca.—In the treatment practised by ordinary physicians in these cases of epilepsy, bromides are very unsatisfactory. I have come to the conclusion, after a period of thirty-five years in trying to cure, or trying to get rid of epilepsy, that the less bromides you give, the better. They seem to keep the fits back till they have accumulated something like the electricity in a Leyden jar, and then they begin to go off and they will have twenty-five or thirty right along and exhaust the patient. A young gentleman from Florida that I had treated about twenty-two years ago, and had not been at home since I treated him. He was having epilepsy; had it while he was here. I wanted him to go up to the Craig Colony and have a talk with one of the physicians while he was here. He said he had learned all about his epileptic case that he wished to know from doctors. He said, "I know when they are coming on and they go off after a little while." Said he, "if I should take bromide, I could go four months or five months without any fits, then they are awful; I know nothing for several days; I have ceased any treatment of that sort." Now he is able to do all his work and it is more than twenty years since he came to me, and I used to give him bromide. He has never had his faculties disturbed one particle. All the epilepsy he has had has never disturbed his faculties. He keeps a double entry set of books.

Dr. CREGO, Erie.—This reminds me very much of a man driving an automobile. A fellow pulls the lever and if he steers just right, it will go all right; but if he don't steer just right, it is likely to go up a tree or in the river or any place like that. So, if you give bromides, according to Dr. Clark's ideas, you will have success in treating these cases. You must give tonics, you must find out the normal quantity that you want to give this certain patient. If you give him bromides hap-hazard, you won't have any results.

In addition to the remedy which the doctor passed around first, I usually use bromide of zinc, one-eighth of a grain, in addition to the other bromide, and get very good results in the treatment of a certain class of patients. I don't know as I can say exactly what class of cases I give that to, but I have an idea of my own and I can tell the cases where I give one bromide or the other bromide or two bromides. I don't know whether Dr. Clark has the same idea or not, that the character of the epilepsy or the convulsion, or something in the study of the case, gives one the idea, whether to give just one bromide or several, or a combination. If you use these remedies intelligently, you will have results in about 30 per cent. of all your cases, if not 50. If you continue the remedy two years after the last attack in a sufficient dose, I think, in about 30 to 50 per cent. in all cases of epilepsy, where there is no depression of the skull, or no mal-formation, you will have a recovery, if you pursue the treatment correctly.

Dr. CHEESMAN, Cayuga.—I would like to ask Dr. Clark if he is having success in operating for epilepsy, or if he has done any such work at the asylum?

Dr. CLARK.—I would say in answer to the last question, we have done a number of trephinings, but we have found, in a great majority of cases, that it simply relieves the intracranial pressure and consequently a slight cessation from seizures for a time may occur. Unless additional medical treatment is employed, I don't believe trephining or operation for epilepsy would be of value. The additional attention needed is from the physician. Too often the surgeon is impatient. He performs the operation, and as soon as the case recovers from that, he sends it away, just at the time when they need most attention. I think, as Dr. Crego said, that one can get in the habit of finding out whether the patient needs the one bromide, the two bromides or three bromides, by some sort of an intuition. I suppose that comes from seeing a great number of cases.

I wish to call your attention to the fact that I have the photograph of a girl who has been taking 480 grains of bromide a day for over two years, and has changed from having 300 or 400 seizures a month, to that of having none; slight losses of consciousness occur infrequently. In addition to that fact, the bromides have not destroyed the physique, but the patient has grown five or six inches taller, has gained in weight and grown less anemic under the bromide. If this patient were not given the attention which I maintain is absolutely essential to the high doses of bromide, we would not have these excellent effects from the bromide in this case.

